# Supplementary material for: Cryo‐EM structures of tau filaments from SH‐SY5Y cells seeded with brain extracts from cases of Alzheimer's disease and corticobasal degeneration
Source: FEBS Open Bio. 2023 Jul 7;13(8):1394–404. doi: 10.1002/2211-5463.13657 (PMC10392052; doi:10.1002/2211-5463.13657)
Supplement: Supplementary file 1 — Fig. S1. Cryo‐EM structure determination. a‐b. Representative micrographs of tau filaments from SH‐SY5Y cells seeded with filaments from Alzheimer's disease and corticobasal degeneration, respectively. Scale bar, 50 nm. c‐d. Side views of reconstructed density of tau filaments from SH‐SY5Y cells seeded with filaments from Alzheimer's disease and corticobasal degeneration, respectively. Scale bar, 2 nm. e‐f. Fourier shell correlation (FSC) curves for tau filaments from SH‐SY5Y cells seeded with filaments from Alzheimer's disease and corticobasal degeneration, respectively. Solvent‐corrected FSC curves between independently refined half‐maps are shown in black; FSC curves between the refined model and the reconstruction from all particles are shown in red; FSC curves between a model refined against half map 1 against half map 1 are shown in dashed yellow; FSC curves of the same model against half map 2 are shown in blue. In panels d and f, the seeded structure type 1 is shown above type 2. Fig. S2. Immunohistochemistry and immuno‐blotting of AD and CBD cases. a. Immunostaining of sections from occipital cortex of the Alzheimer's disease case and putamen of the corticobasal dewgeneration case using anti‐tau antibodies AT8, RD3, Anti‐4R and RD4. Scale bar, 25 mm. b. Immunoblotting of sarkosyl‐insoluble fractions from occipital cortex of the Alzheimer's disease case and putamen of the corticobasal degeneration case using anti‐tau antibodies T46, RD3 and Anti‐4R. Fig. S3. Aβ42 levels in AD and CBD. Aβ42 levels in sarkosyl‐insoluble fractions from AD and CBD were determined by sandwich ELISA. The results are expressed as means ± S.E.M. (n = 4‐8). Fig. S4. Immunoblotting and immunoelectron microscopy of tau assemblies from the sarkosyl‐insoluble fractions of SH‐SY5Y cells. a. Schematic of the seeded tau aggregation model. b. Immunoblotting of sarkosyl‐insoluble tau from seeded SH‐SY5Y cells using anti‐HA and pS396 tau antibodies. Total tau was detected in the sarkosyl‐solub [file FEB4-13-1394-s001.docx]

**Supplementary information**

**Cryo-EM structures of tau filaments from SH-SY5Y cells seeded with brain extracts from cases of Alzheimer’s disease and corticobasal degeneration**

Airi Tarutani^1,2^*, Sofia Lövestam^3^*, Xianjun Zhang^4^, Abhay Kotecha^4^, Andrew C. Robinson^5^, David M.A. Mann^5^, Yuko Saito^6^, Shigeo Murayama^6,7^, Taisuke Tomita^2^, Michel Goedert^3@^, Sjors H.W. Scheres^3@^, Masato Hasegawa^1@^

^1^ Department of Brain and Neuroscience, Tokyo Metropolitan Institute of Medical

Science, Tokyo, Japan

^2^ Laboratory of Neuropathology and Neuroscience, Graduate School of

Pharmaceutical Sciences, The University of Tokyo, Tokyo, Japan

^3^ Medical Research Council Laboratory of Molecular Biology, Cambridge, UK

^4^ Thermo Fisher Scientific, Eindhoven, The Netherlands

^5^ School of Biological Sciences, University of Manchester, Salford, UK

^6^ Department of Neuropathology, Tokyo Metropolitan Institute of Gerontology, Tokyo, Japan

^7^ Brain Bank for Neurodevelopmental, Neurological and Psychiatric Disorders, United Graduate School of Child Development, Osaka University, Osaka, Japan

* These authors contributed equally

^@^ Correspondence to: mg@mrc-lmb.cam.ac.uk; scheres@mrc-lmb.cam.uk;

hasegawa-ms@igakuken.or.jp

**
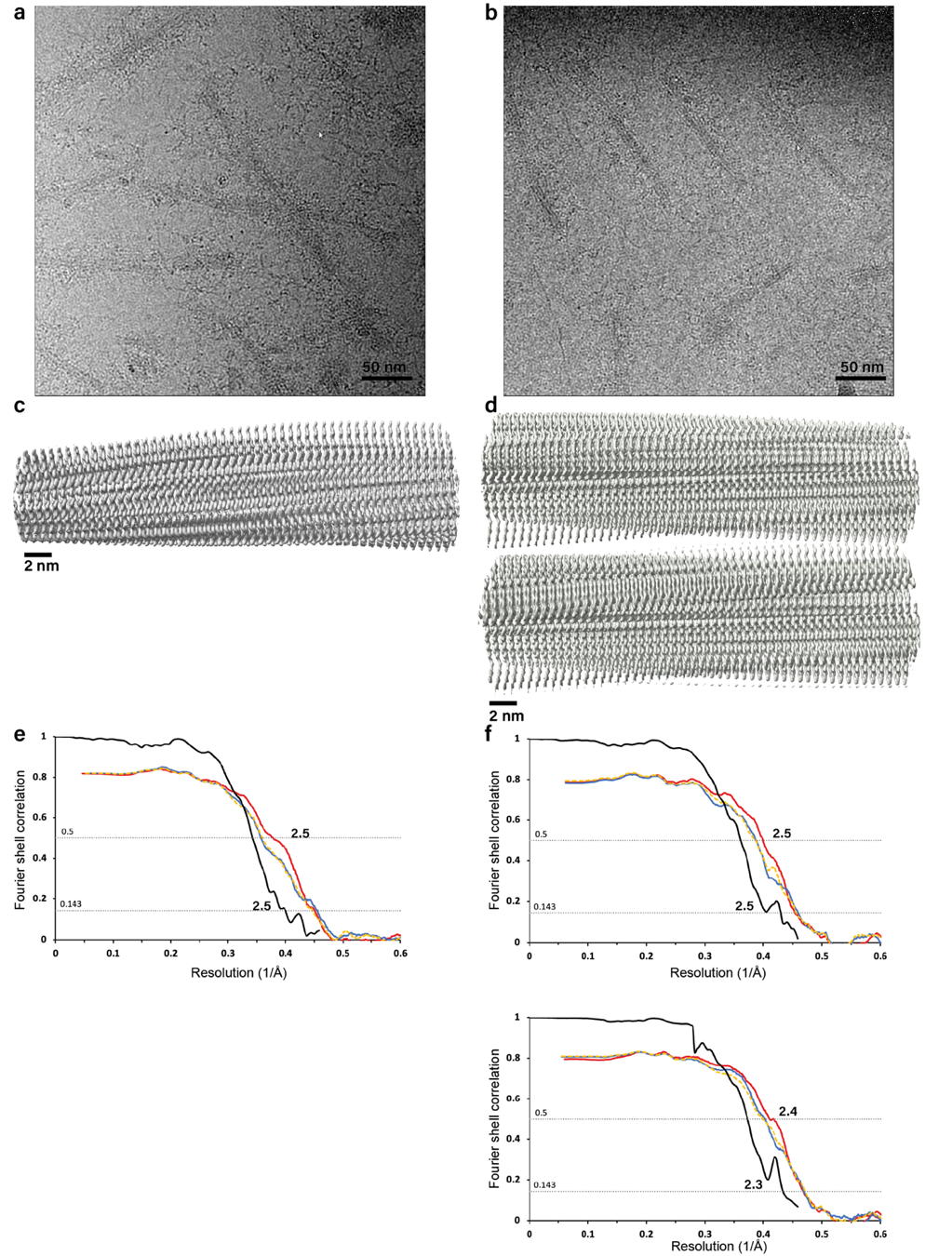
**

**Supplementary Figure 1: Cryo-EM structure determination.**

**a-b.** Representative micrographs of tau filaments from SH-SY5Y cells seeded with filaments from Alzheimer’s disease and corticobasal degeneration, respectively. Scale bar, 50 nm. **c-d.** Side views of reconstructed density of tau filaments from SH-SY5Y cells seeded with filaments from Alzheimer’s disease and corticobasal degeneration, respectively. Scale bar, 2 nm. **e-f.** Fourier shell correlation (FSC) curves for tau filaments from SH-SY5Y cells seeded with filaments from Alzheimer’s disease and corticobasal degeneration, respectively. Solvent-corrected FSC curves between independently refined half-maps are shown in black; FSC curves between the refined model and the reconstruction from all particles are shown in red; FSC curves between a model refined against half map 1 against half map 1 are shown in dashed yellow; FSC curves of the same model against half map 2 are shown in blue. In panels d and f, the seeded structure type 1 is shown above type 2.

**
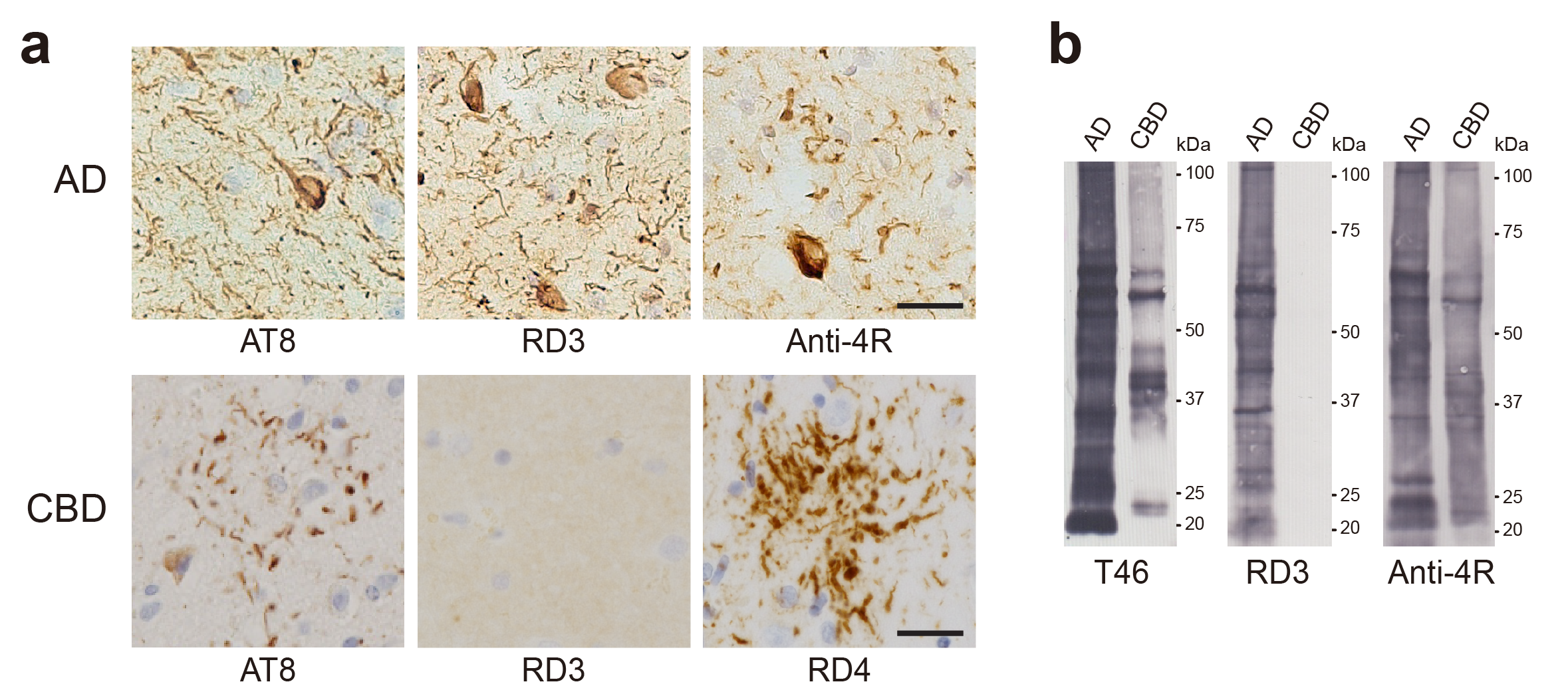
**

**Supplementary Figure 2: Immunohistochemistry and immuno-blotting of AD and CBD cases.**

**a.** Immunostaining of sections from occipital cortex of the Alzheimer’s disease case and putamen of the corticobasal dewgeneration case using anti-tau antibodies AT8, RD3, Anti-4R and RD4. Scale bar, 25 mm. **b.** Immunoblotting of sarkosyl-insoluble fractions from occipital cortex of the Alzheimer’s disease case and putamen of the corticobasal degeneration case using anti-tau antibodies T46, RD3 and Anti-4R.


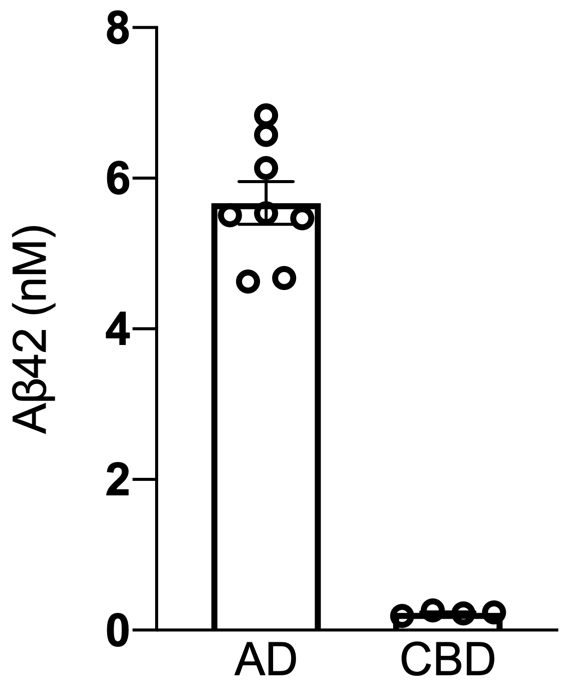


**Supplementary Figure 3: Aβ42 levels in AD and CBD**

Aβ42 levels in sarkosyl-insoluble fractions from AD and CBD were determined by sandwich ELISA. The results are expressed as means ± S.E.M. (n=4-8).

**
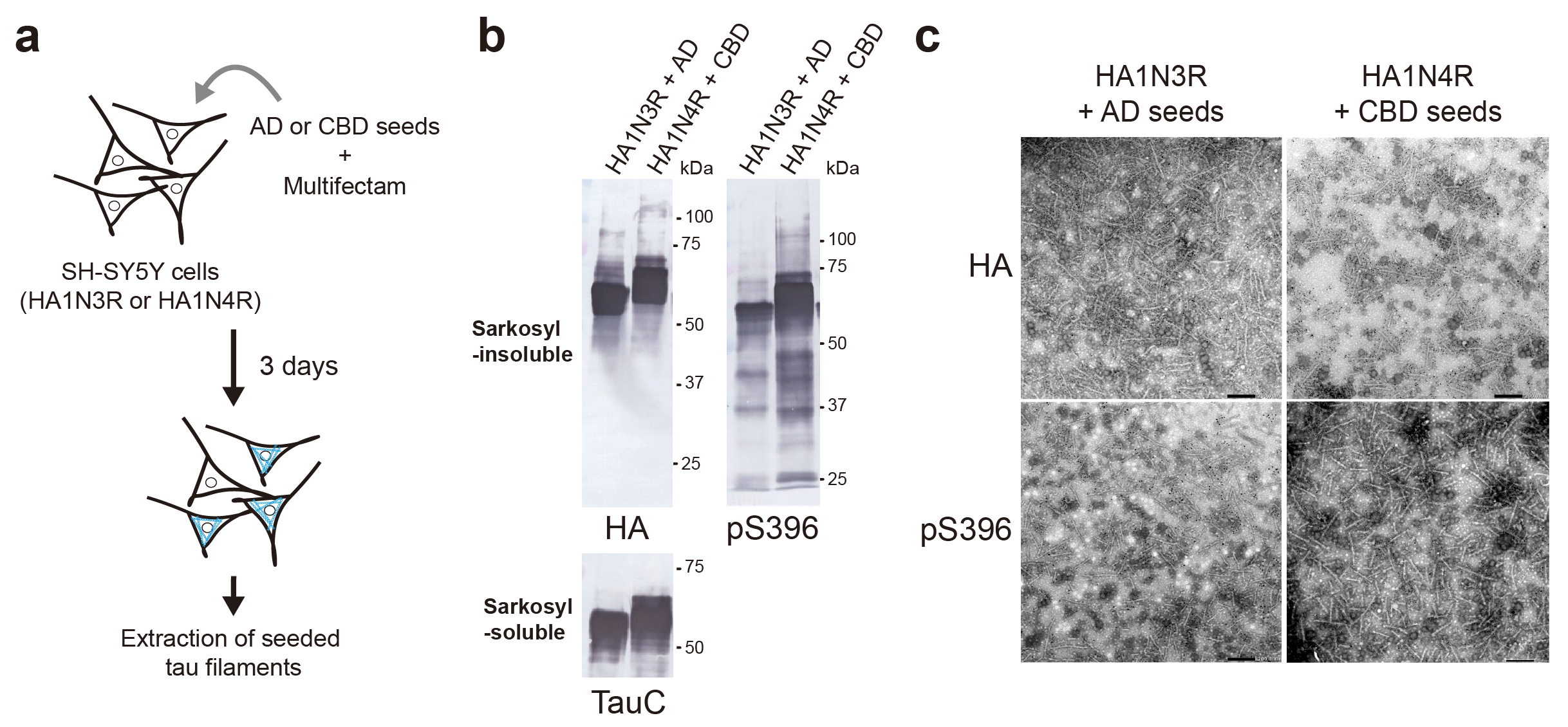
**

**Supplementary Figure 4: Immunoblotting and immunoelectron microscopy of tau assemblies from the sarkosyl-insoluble fractions of SH-SY5Y cells.**

**a.** Schematic of the seeded tau aggregation model. **b.** Immunoblotting of sarkosyl-insoluble tau from seeded SH-SY5Y cells using anti-HA and pS396 tau antibodies. Total tau was detected in the sarkosyl-soluble fraction using anti-tau antibody TauC. **c.** Immunoelectron microscopy of sarkosyl-insoluble tau frojm seeded SH-SY5Y cells using anti-HA and anti-pS396 tau antibodies. The secondary antibody was conjugated to 5 nm gold particles. Scale bar, 200 nm.

**Supplementary Table 1** Cryo-EM data acquisition and model refinement statistics

| **Titan Krios G3** | **SH-SY5Y**  **1N3R**  (EMDB-17121)  (PDB 8ORE) | **SH-SY5Y 1N4R type 1**  (EMDB-17122)  (PDB 8ORF) | **SH-SY5Y 1N4R type 2**  (EMDB-17123)  (PDB 8ORG) |
| --- | --- | --- | --- |
| **Data acquisition** |  |  |  |
| Electron gun | CFEG | CFEG | CFEG |
| Detector | Falcon 4 | Falcon 4 | Falcon 4 |
| Energy filter slit (eV) | 10 | 10 | 10 |
| Magnification | 165,000 | 165,000 | 165,000 |
| Voltage (kV) | 300 | 300 | 300 |
| Electron dose (e-/Å^2^) | 40 | 40 | 40 |
| Defocus range (μM) | 0.5 to 2.5 | 0.5 to 2.5 | 0.5 to 2.5 |
| Pixel size (Å) | 0.727 | 0.727 | 0.727 |
| **Data processing** |  |  |  |
| Initial particle images (no.) | 146,442 | 218,385 | 218,385 |
| Final particle images (no.) | 16,052 | 35,617 | 75,380 |
| Helical twist (º) | -1.48 | -0.84 | -0.92 |
| Helical rise (Å) | 4.75 | 4.75 | 4.75 |
| Symmetry imposed | na | na | na |
| Map resolution FSC 0.143 (Å) | 2.5 | 2.5 | 2.3 |
| **Refinement** |  |  |  |
| Initial model used (PDB code) | 7qkk | 6tjx | 6tjx |
| Model resolution FSC 0.5 (Å) | 2.5 | 2.5 | 2.4 |
| Map sharpening *B* factor (Å^2^) | -28.31 | -24.20 | -26.89 |
| Model composition  Non-hydrogen atoms  Protein residues  Ligands | 1770  231  0 | 2271  303  0 | 1941  258  0 |
| *B* factors (Å^2^)  Protein  Ligand | 72.28  na | 88.16  na | 77.56  na |
| R.m.s. deviations  Bond lengths (Å)  Bond angles (°) | 0.01  2.179 | 0.01  1.272 | 0.01  1.263 |
| Validation  MolProbity score  Clashscore  Poor rotamers (%) | 1.17  0.55  0 | 0.85  0  0 | 0.58  0  0 |
| Ramachandran plot  Favored (%)  Allowed (%)  Disallowed (%) | 92.00  8.00  0 | 94.95  5.05  0 | 97.62  2.38  0 |
